# Impacts of Heart Failure and Physical Performance on Long-Term Mortality in Old Patients With Chronic Kidney Disease

**DOI:** 10.3389/fcvm.2021.680098

**Published:** 2021-06-04

**Authors:** Shuo-Chun Weng, Yu-Chi Chen, Chiann-Yi Hsu, Chu-Sheng Lin, Der-Cherng Tarng, Shih-Yi Lin

**Affiliations:** ^1^Institute of Clinical Medicine, School of Medicine, College of Medicine, National Yang Ming Chiao Tung University, Taipei, Taiwan; ^2^Division of Nephrology, Department of Internal Medicine, Center for Geriatrics and Gerontology, Taichung Veterans General Hospital, Taichung, Taiwan; ^3^Institute of Clinical Nursing, College of Nursing, National Yang Ming Chiao Tung University, Taipei, Taiwan; ^4^Biostatistics Task Force of Taichung Veterans General Hospital, Taichung, Taiwan; ^5^Department of Family Medicine, Center for Geriatrics and Gerontology, Taichung Veterans General Hospital, Taichung, Taiwan; ^6^Department and Institute of Physiology, National Yang Ming Chiao Tung University, Taipei, Taiwan; ^7^Division of Nephrology, Department of Medicine, Taipei Veterans General Hospital, Taipei, Taiwan; ^8^Department of Biological Science and Technology, Center for Intelligent Drug Systems and Smart Bio-Devices (IDS2B), College of Biological Science and Technology, National Yang Ming Chiao Tung, Hsinchu, Taiwan; ^9^Division of Endocrinology and Metabolism, Department of Internal Medicine, Center for Geriatrics and Gerontology, Taichung Veterans General Hospital, Taichung, Taiwan

**Keywords:** comprehensive geriatric assessment, ejection fraction, handgrip strength, mortality, physical functionality, timed up-and-go test

## Abstract

**Background:** In patients with chronic kidney disease (CKD), physical functional limitations and heart failure (HF) are common, and each is associated with adverse outcomes. However, their joint effects on mortality are not clear.

**Design and Methods:** Using administration data from the geriatric department in a tertiary hospital, retrospective longitudinal analyses of patients aged ≥65 years with CKD were consecutively enrolled from February 2010 to November 2015. Baseline CKD stages, HF with reduced and preserved ejection fraction (HFrEF and HFpEF), Rockwood frailty index, handgrip strength (HGS), 6-m walking speed, and timed up-and-go test were used to predict the prevalence of frailty, physical disability, and all-cause mortality.

**Results:** Among 331 old patients with CKD, their mean age was 81.3 ± 6.6 years. CKD stages showed the following distributions: stage 3, 74.9%; stage 4, 15.7%; stage 5, 9.4%. The prevalence of HF was 23.3%, and Rockwood frailty was 74.3%. Rockwood frailty and HF were both significantly associated with CKD stages. After a mean follow-up period of 3.1 ± 2.1 years, 44 patients died, and a crude analysis showed that stage 4, stage 5 CKD, low HGS, and Rockwood frailty index were associated with mortality. Regarding the survival of these patients, the adjusted mortality hazard ratio for CKD stage 5 was 3.84 against stage 3A [95% confidence interval (CI) 1.51–9.75], 1.04 (95% CI 1.01–1.07) for higher Rockwood frailty score, 4.78 (95% CI 1.26–18.11) for HFrEF, and 3.47 (95% CI 1.15–10.42) for low HGS. Survival analysis using Kaplan–Meier survival plots showed that patients with both HF and poor HGS had the poorest survival.

**Conclusions:** Our study shows that both low physical performance and HF were common in old CKD patients and were associated with CKD stages. HF, frailty, and HGS all independently predicted the mortality of these CKD patients. The mortality is especially high amongst individuals with both HF and decreased HGS.

## Introduction

Chronic kidney disease (CKD) is a public health problem worldwide, especially in older populations. CKD has prevalence ranging from 23.4 to 35.8% based on a systematic review of population-based studies (1). The National Health and Nutrition Examination Survey (NHANES 2007–2012) reported a CKD prevalence of 33.2% for those aged ≥60 years (2). Individuals with CKD have a mortality rate double that of the general population, and more than half of the deaths in these patients are from cardiovascular disease (CVD). CVD in CKD patients includes the following: coronary artery disease (CAD), acute myocardial infarction (MI), heart failure (HF), valvular heart disease, cerebrovascular accidents (CVA), peripheral artery disease (PAD), thromboembolic disease, and sudden cardiac death with HF being the leading cause (3). The study on Atherosclerosis Risk in Communities (ARIC) reports a threefold higher risk of incident HF in those with estimated glomerular filtration rate (eGFR) <60 mL/1.73m^2^/min compared with those with eGFR >90 mL/min/1.73m^2^. Both the prevalence and incidence of HF also increase with the severity of CKD. CKD patients develop HF with reduced (HFrEF) and preserved ejection fraction (HFpEF), but HFpEF is more common (4, 5).

It has been proposed that there is close interdependence between CKD and chronic HF (CHF), and their common pathophysiologic pathways can lead to function deterioration and lower life expectancy (6, 7). Our previous study reports that low eGFR is potentially and highly capable of distinguishing between deaths in all patients and deaths in HF patients (8). Based on the close relationship between CHF and CKD, individualized treatments for patients with chronic systolic HF are applied according to different stages of CKD (6). In addition to HF, CKD also has a negative impact on physical functions and frailty. Dalrymple et al. report a 24% prevalent frailty in those patients with eGFR <45 mL/1.73 m^2^/min (9). In dialysis-dependent CKD patients, the frailty prevalence is >60%, which is independently linked with adverse clinical outcomes, including mortality and repetitive hospitalization for all stages of CKD (9).

Comprehensive geriatric assessment (CGA), a multidimensional diagnostic tool for determining the medical, psychological, and functional capabilities in the frail population, can predict prognosis for hospitalized old patients with HF (10). Apart from these, walking speed (WS) (11) and the timed up-and-go (TUG) test, the other two indicators for frailty, are associated with quality of life and progress of NYHA functional class in patients with congestive HF (12). Only a few studies have yet investigated the joint effects of physical disability and HF on outcomes in old patients with CKD (13).

Given the close relationship and adverse outcomes of CHF and frailty in CKD, a better understanding of their individual and combined impacts is important for planning interventions to improve prognosis and reduce healthcare costs. In our present study on old patients with CKD, we aimed to use CGA to evaluate the prevalence of frailty and determine the independent and composite prognostic effects of physical functions, HFpEF, and HFrEF on all-cause mortality in old patients with CKD.

## Materials and Methods

### Study Participants

Our study was conducted in a medical center Taichung Veterans General Hospital (TCVGH), between February 2, 2010, and November 26, 2015, based on the records of the case management care system of the Hospital's Center for Geriatrics and Gerontology. The study was approved by the institutional review board of TCVGH (No.CF20293). Participating patients were eligible if they had visited the inpatient and outpatient clinics of the geriatric department. The inclusion criteria were age ≥65 years, without complicated neurologic disorders, with a diagnosis of CKD with or without HF. After enrollment, medical history, including basic personal information (age, gender, history of chronic illness, education, and source of referral) was recorded.

### Diagnosis of HF and CKD

CHF was defined by the International Classification of Diseases, 9th Revision, Clinical Modification (ICD-9-CM) codes (428.0-428.9, 402.91). Besides this, a 2-D echocardiogram (ECHO) and N-terminal pro-B-type natriuretic peptide (NT-proBNP) were obtained to diagnose and differentiate HFpEF and HFrEF under a standard protocol (ASE or EACVI protocol) (14–16). By 2-D ECHO, LVEF ≥ 50% was defined as HFpEF and <50% as HFrEF according to the American College of Cardiology Foundation/American Heart Association guideline (17). Other codes for relevant diseases were atrial fibrillation (AF) 427.31, cardiac arrhythmia 427.0–427.9, and CKD 585.XX.

CKD patients were confirmed by the following criteria: eGFR <60 ml/1.73m^2^/min, urine albumin/creatinine ratio (ACR) >30 mg/g (18), urine protein/creatinine (PC) ratio >0.2 mg/g (19), or showing abnormal kidney images. Concomitant medication data associated with HF were extracted according to the anatomic therapeutic chemical (ATC) codes. Comorbid conditions were measured using the Charlson comorbidity index (CCI), which includes 19 chronic diseases weighted based on their associations with mortality (20) but with some modifications as neither HF nor CKD are considered within the CCI (10). Finally, clinical records were selected for the 331 enrolled patients concurrent with 2-D ECHO examinations (254 non-HF and 77 HF patients with 45 HFpEF and 32 HFrEF) and CKD (147 stage 3A, 101 stage 3B, 52 stage 4, and 31 stage 5 CKD patients).

### CGA and Evaluation of Physical Functionality

Trained research nurses administered a CGA with standardized measures, of which the components were previously described (21), including the mini-nutritional assessment (MNA) used to identify old adults at risk for malnutrition (22). HGS was measured by a dynamometer (Smedley's Dynamometer, TTM, Tokyo, Japan), and slowness was measured by the 6-m WS (6MW). For the TUG test, participants were to stand up from a 46-cm-high armchair with back support, walk in a straight line for 3 m, turn around, walk back to the chair, and sit down as quickly and safely as possible (23). The timing started when the investigator said “go” and stopped when the participant sat back down on the chair. Arbitrary cutoff points instead of traditional values were used to define frailty parameters, including TUG, HGS, and 6MW (8, 24). TUG values were separated into quartiles, and the Chi-square test was used to determine the appropriateness of 24 s. The HGS values were divided into tertiles, and the cutoff point was 20.4 kg for men and 15.435 kg for women. Values of 6MW were calculated as deciles with a cutoff point of 22 s for men and 30 s for women.

### Frailty Index

Frailty was defined according to the Asia-Pacific clinical practice guideline (24). A modified Rockwood frailty index (25) was used to measure frailty by utilizing health deficits collected in health assessments, including 11 chronic diseases, four items (MNA-SF, TUG, HGS, 6MW) of CGA, and five abnormal laboratory data. Categories were generated according to established cutoffs in community-dwelling cohorts to match the Fried physical phenotype: non-frail (0–0.1), pre-frail (>0.1–0.21), and frail (>0.21) (26, 27).

### Study Outcome and Follow-Up

The index date was the date of HF and CKD diagnosis. CGA and 2-D ECHO were completed around the time of HF diagnosis. The patient outcome was all-cause mortality obtained from the Clinical Information Research and Development Center, TCVGH, and the accuracy of death was validated by Taiwan's National Death Registry according to the ICD-9 (ICD9 001.x-999.x) or ICD10 (A00.x-Z99.x). All participants were followed up until death or June 19, 2018, to prevent lead-time bias.

### Statistical Analyses

For continuous variables, we used the Kolmogorov–Smirnov test to test the normality of sample distributions. Continuous variables were analyzed by the Kruskal–Wallis (more than a dichotomy) and Mann–Whitney *U* (dichotomy) tests, generating the median and interquartile range (IQR). Categorical variables, expressed as percentages, were tested by chi-square or Fisher's exact test. All-cause mortality was delineated based on previously defined high or low functioning status, the severity of HF, and stages of CKD. Then, Cox proportional hazard models were finally applied in the multivariate analyses to estimate the hazard ratios of study outcomes after adjusting for age and gender. To determine cumulative effects of HF with preserved or reduced EF, Rockwood frailty, and physical functionality (HGS, TUG, 6MW) on survival, they were stratified into subgroups according to the cutoff values (8). Kaplan–Meier (KM) plots were generated to estimate the cumulative survival rate in different subgroups by log rank (Mantel–Cox) and pairwise comparison to judge which entity displayed significance; *p*-values for non-linearity were calculated using the null hypothesis test. Statistical significance was set at *p* < 0.05. Statistical analyses were performed with the SPSS for Windows version 22.0 (SPSS Institute Inc., Chicago, USA).

## Results

### Clinical Characteristics of Patients

The mean age of the 331 CKD patients was 81.3 ± 6.6 years with a mean follow-up period of 3.1 ± 2.1 years. The percentage of CKD stage 3, 4, or 5 was 74.9, 15.7, or 9.4%, respectively. Among them, patients from CKD stage 3A to 5 had similar distributions for the following: gender, body mass index (BMI), CCI, LVEF, cardiac arrhythmia, β-blocker, angiotensin-converting enzyme inhibitor (ACEI) or angiotensin II receptor blockers (ARB), and digoxin except for distribution of age, HF, HFpEF, HFrEF, NT-proBNP, low-density lipoprotein (LDL), albumin, glycated hemoglobin (Hba1c), eGFR, proteinuria, mineralocorticoid receptor antagonist (MRA), and anticoagulants (Table 1).

**Table 1:** Baseline characteristics of older patients with different staging of CKD.

**Characteristics**	**CKD stage 3A (*n* = 147)**	**CKD stage 3B (*n* = 101)**	**CKD stage 4 (*n* = 52)**	**CKD stage 5 (*n* = 31)**	***p*-value**
Age, years	83.1 (77.1–86.3)	81.8 (76.0–86.7)	83.2 (79.0–87.1)	78.8 (73.3–83.6)	0.043
Male	101 (68.7)	71 (70.3)	39 (75.0)	21 (67.7)	0.846
BMI, kg/m^2^	24.6 (22.6–27.3)	24.5 (22.1–28.2)	24.0 (21.8–27.0)	23.8 (21.2–26.1)	0.275
**Heart condition**					0.001
Non–heart failure	119 (81.0)	84 (83.2)	36 (69.2)	15 (48.4)	
HFpEF	15 (10.2)	12 (11.9)	10 (19.2)	8 (25.8)	
HFrEF	13 (8.8)	5 (4.9)	6 (11.5)	8 (25.8)	
Atrial fibrillation	6 (4.1)	2 (2.0)	3 (5.8)	4 (12.9)	0.080
CCI	2.0 (1.0–2.0)	1.0 (1.0–2.0)	1.0 (1.0–2.0)	2.0 (1.0–3.5)	0.747
LVEF	59.0 (54.0–60.0)	58.0 (55.5–62.0)	56.5 (52.0–61.8)	59.0 (49.8–60.3)	0.742
Cardiac arrhythmia	10 (6.8)	6 (5.9)	4 (7.7)	6 (19.4)	0.094
**Laboratory data**					
NT-proBNP, pg/mL	670.6 (366.0–4,119.0)	1,780.0 (337.2–3,925.5)	3,495.0 (1,162.0–16,312.5)	9,150.0 (1,610.0–33,050.0)	0.001
LDL, mg/dL	98.0 (82.0–121.0)	103.5 (81.3–119.0)	87.0 (73.0–108.0)	79.5 (58.0–121.8)	0.037
Albumin, g/dL	4.0 (3.7–4.3)	3.9 (3.3–4.2)	3.8 (3.5–4.1)	3.6 (3.0–3.9)	0.001
Hba1c, %	6.1 (5.7–7.0)	6.3 (5.8–7.0)	6.2 (5.6–7.4)	5.6 (5.2–6.5)	0.010
**Medications**					
Diuretics	108 (73.5)	72 (71.3)	47 (90.4)	24 (77.4)	0.054
MRA	31 (21.1)	16 (15.8)	18 (34.6)	15 (48.4)	0.001
β-blocker	88 (59.9)	60 (59.4)	41 (78.9)	20 (64.5)	0.078
ACEI or ARB	110 (74.8)	77 (76.2)	43 (82.7)	19 (61.3)	0.183
Anti-platelet agents	95 (64.6)	66 (65.4)	44 (84.6)	21 (67.7)	0.052
Anti-coagulants	24 (16.3)	13 (12.9)	19 (36.5)	6 (19.4)	0.003
Digoxin	17 (11.6)	11 (10.9)	10 (19.2)	7 (22.6)	0.197

*Continuous data were expressed as median (IQR) and analyzed by the Kruskal–Wallis test. Categorical data were expressed as number and percentage and analyzed by the Chi-Square test. CKD, chronic kidney disease; BMI, body mass index; HFpEF, heart failure with preserved ejection fraction; HFrEF, heart failure with reduced ejection fraction; CCI, Charlson Comorbidity Index; IQR, interquartile range; LVEF, left ventricular ejection fraction; NT-proBNP, N-terminal pro-B-type natriuretic peptide; LDL, low-density lipoprotein; Hba1c, glycated hemoglobin; eGFR, estimated glomerular filtration rate; MRA, mineralocorticoid receptor antagonist; ACEI, angiotensin-converting enzyme inhibitor; ARB, angiotensin II receptor blockers; eGFR, calculated by using modified Modification diet of renal disease (MDRD) formula, was utilized to evaluate renal function*.

### Frailty and Physical Performance in CKD Patients

The Rockwood frailty assessment showed 74.3% of CKD patients were frail. Compared with patients of CKD stages 3A and 3B, CKD patients with stages 4 and 5 showed lower scores of MNA-short form (MNA-SF) but higher scores on the Rockwood index and showed no differences with the TUG and HGS. The Rockwood frailty index was significantly associated with CKD stages (Table 2).

**Table 2:** Baseline physical functionality in older patients with CKD.

**Characteristics**	**CKD stage 3A (*n* = 147)**	**CKD stage 3B (*n* = 101)**	**CKD stage 4 (*n* = 52)**	**CKD stage 5 (*n* = 31)**	***p*-value**
MNA-SF (0–14)	13.0 (11.0–14.0)	13.0 (12.0–14.0)	12.5 (10.3–14.0)	12.0 (9.0–14.0)	0.025
Timed up-and-go test, sec	17.0 (13.0–24.0)	18.0 (13.0–24.0)	20.0 (15.3–26.0)	16.0 (13.0–24.0)	0.339
TUG, s ≥24	39 (26.5)	27 (26.7)	17 (32.7)	8 (25.8)	0.838
Handgrip strength, kg	21.5 (14.5–25.2)	18.0 (14.1–24.5)	21.8 (18.6–25.5)	17.7 (14.5–24.9)	0.622
HGS, kg					0.461
F ≤ 15.435/M ≤ 20.4	59 (40.1)	55 (54.5)	22 (42.3)	17 (54.8)	
Rockwood frailty index	26.7 (17.7–35.3)	29.4 (21.1–34.3)	29.7 (23.2–41.2)	36.8 (23.5–47.1)	0.003
Non-frail	9 (6.1)	4 (4.0)	1 (1.9)	3 (9.7)	0.233
Pre-frail	36 (24.5)	20 (19.8)	10 (19.2)	2 (6.5)	
Frail	102 (69.4)	77 (76.2)	41 (78.8)	26 (83.9)	

*Continuous data were expressed as median (IQR) and analyzed by the Kruskal–Wallis test. Categorical data were expressed as number and percentage and analyzed by the Chi-Square test. CKD, chronic kidney disease; MNA-SF, mini-nutritional assessment-short form; TUG, Timed Up-and-Go test; HGS, handgrip strength*.

### Heart Failure, HFpEF, and HFrEF in the CKD Patients

In all these patients, the prevalence of HF, HFpEF, and HFrEF were 23.3, 13.6, and 9.7%, respectively. Compared with non-HF patients, HF patients in the CKD cohort had higher percentages in the following: CVD, AF, MI, cardiac arrhythmia, lower LVEF, and lower eGFR (data not shown). Patients with HF and CKD had significantly high Rockwood frailty scores (*p* < 0.001) but a marginally higher percentage of longer TUG test with cutoff ≥24 s (*p* = 0.065).

Due to possibly mixing HFpEF and HFrEF in a mutual effect on the endpoints, we differentiated between these two entities (Table 3). In the geriatric assessment, lower HGS and longer TUG time were observed in the CKD patients with HFrEF although of no statistical significance. The Rockwood frailty index scores were significantly increased in the CKD patients with HFrEF.

**Table 3:** Comprehensive geriatric assessment in older CKD patients with and without heart failure.

**Characteristics**	**Non-heart failure (*n* = 254)**	**HFpEF (*n* = 45)**	**HFrEF (*n* = 32)**	***p*-value**
Age, years	82.8 (76.4–86.5)	81.2 (77.2–85.6)	82.4 (76.5–86.9)	0.980
Male	174 (68.5)	34 (75.6)	24 (75.0)	0.518
BMI	24.3 (22.0–27.2)	24.3 (22.8–26.6)	24.5 (22.1–28.8)	0.533
CCI	1.0 (1.0–2.0)	2.0 (1.0–2.5)	2.0 (1.0–2.3)	0.641
LVEF	60.0 (56.0–62.0)	59.0 (56.3–60.8)	42.0 (31.0–49.0)	<0.001
Cardiac arrhythmia	12 (4.7)	10 (22.2)	4 (12.5)	<0.001
Atrial fibrillation	3 (1.2)	9 (20.0)	3 (9.4)	<0.001
**Geriatric assessment**				
MNA-SF (0–14)	13.0 (11.0–14.0)	13.0 (12.0–14.0)	12.5 (10.0–13.0)	0.053
Timed up-and-go test, sec	18.0 (13.8–23.3)	17.0 (12.0–27.5)	18.5 (15.3–29.5)	0.126
Timed up-and-go test ≥24, s	63 (24.8)	15 (33.3)	13 (40.6)	0.108
Handgrip strength, kg	20.5 (14.2–25.0)	23.0 (15.3–27.3)	17.1 (14.3–19.2)	0.332
**Handgrip strength, kg**				
F ≤ 15.435/M ≤ 20.4	110 (43.3)	20 (44.4)	28 (87.5)	0.054
Rockwood frailty index	25.0 (17.4–33.3)	41.2 (33.3–47.2)	42.1 (33.3–50.0)	<0.001
Non-frail	17 (6.7)	0 (0.0)	0 (0.0)	<0.001
Pre-frail	68 (26.8)	0 (0.0)	0 (0.0)	
Frail	169 (66.5)	45 (100.0)	32 (100.0)	

*Continuous data were expressed as median (IQR) and analyzed by the Mann–Whitney U-test. Categorical data were expressed as number and percentage and analyzed by the Chi-Square test. HFpEF, heart failure with preserved ejection fraction; HFrEF, heart failure with reduced ejection fraction; CCI, Charlson Comorbidity Index; LVEF, left ventricular ejection fraction; MNA-SF, mini-nutritional assessment-short form*.

### Predictors of Survival in Patients With CKD

**Figure 1:**
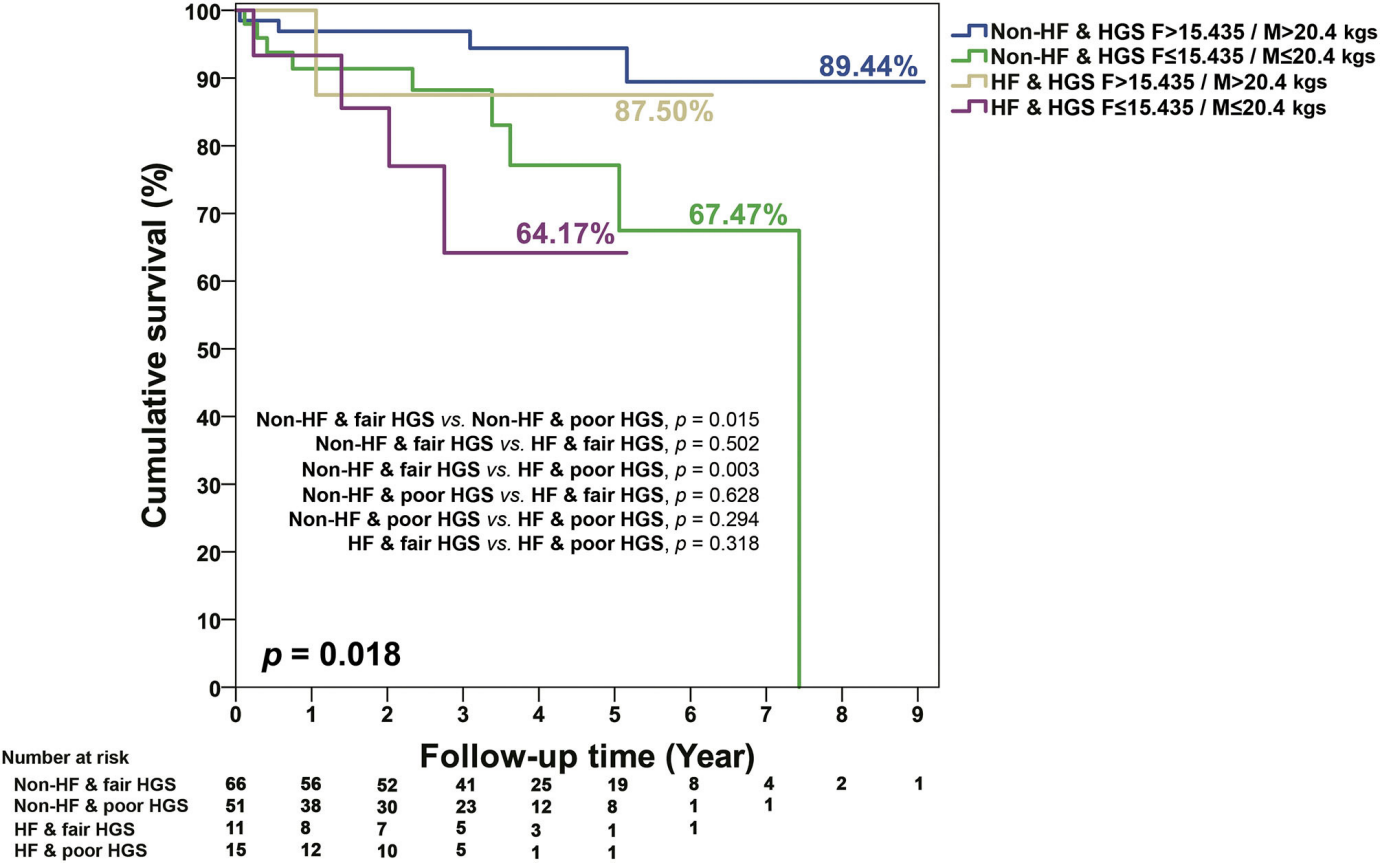
KM survival curves for mortality stratified by the different levels of HGS, HF, and non-HF. Poor HGS in females ≤ 15.435 kg/males ≤ 20.4 kg; fair HGS in females >15.435 kg/males >20.4 kg.

In terms of baseline characteristics of CKD survivors and non-survivors, non-survivors had a relatively longer TUG, significantly poorer HGS, and significantly higher Rockwood frailty scores [median (quartiles) = 34.3 (25.0–43.9) vs. 27.8 (18.8–35.3); Table 4]. In addition, non-survivors in CKD patients also had lower levels of serum LDL, albumin, and eGFR. More of them also received medications of diuretics, MRA, β-blocker, and digoxin (Table 4). When those patients were grouped into the combined HF and frailty status, 77 were HF and frail, none was HF and non-frail, 169 were non-HF and frail, and 85 were non-HF and non-frail (Table 4). During the follow-up period [median (quartiles) = 3.1 (1.1 to 4.7) years], a univariate Cox regression model (Table 5) showed that in CKD stages 4 and 5 compared with stage 3A, the continuous and discrete values of HGS were significantly associated with all-cause mortality. In the multivariate Cox proportional hazards model, poor HGS [model 1, Table 5, adjusted hazard ratio (aHR) = 0.91, 95% CI 0.84–0.99] and HFrEF (aHR = 4.78, 95% CI 1.26–18.11) were significantly associated with all-cause mortality after adjusting for age/gender, a heart condition, and different stages of CKD. When HGS was divided into categorized values (model 2, Table 5), those with low HGS (female ≤ 15.435 kg / male ≤ 20.4 kg) had a significant risk for all-cause death (aHR = 3.47, 95% CI 1.15–10.42). Patients of CKD stage 5, when compared with stage 3A (aHR = 3.84, 95% CI 1.51–9.75) and high Rockwood frailty score (aHR = 1.04, 95% CI 1.01–1.07), were associated with high patient mortality (model 3, Table 5). When patients were stratified into subgroups with and without HF and Rockwood frailty and abnormal physical functionality (HGS, TUG, 6MW), it was shown that HF patients associated with decreased HGS had the poorest survival, followed by non-HF patients with decreased HGS, HF with fair HGS, and non-HF patients with fair HGS, respectively (*p* = 0.018; Figure 1). However, there were no additive effects between HF and frailty, TUG, or 6MW on survival (Supplementary Figures 1A–C).

**Table 4:** Comparison between survivors and non-survivors in older patients with CKD.

	**Alive (*n* = 287)**	**Death (*n* = 44)**	***p*-value**
Age, years	82.6 (76.3–86.2)	83.4 (78.5–87.1)	0.250
Male	201 (70.0)	31 (70.5)	1.000
CKD			0.005
Stage 3A	135 (47.0)	12 (27.3)	
Stage 3B	89 (31.0)	12 (27.3)	
Stage 4	41 (14.3)	11 (25.0)	
Stage 5	22 (7.7)	9 (20.5)	
BMI, kg/m^2^	24.3 (22.2–27.3)	24.5 (21.2–26.8)	0.504
CCI	1.0 (1.0–2.0)	1.5 (1.0–3.0)	0.242
Atrial fibrillation	10 (3.5)	5 (11.4)	0.052
Heart condition			0.525
Non-HF	223 (77.7)	31 (70.5)	
HFpEF	38 (13.2)	7 (15.9)	
HFrEF	26 (9.1)	6 (13.6)	
LVEF	58.0 (54.0–61.0)	58.0 (54.0–60.0)	0.455
**Geriatric assessment**			
MNA-SF (0–14)	13.0 (11.0–14.0)	13.0 (11.0–14.0)	0.807
Timed up-and-go test, sec	17.0 (13.0–24.0)	20.0 (15.3–28.8)	0.080
TUG, s ≥24	76 (26.5)	15 (34.1)	0.383
Handgrip strength, kg	21.2 (14.4–25.6)	17.7 (14.2–20.7)	0.102
HGS, kg			0.034
F ≤ 15.435/M ≤ 20.4	53 (42.4)	13 (72.2)	
Rockwood frailty index	27.8 (18.8–35.3)	34.3 (25.0–43.9)	0.002
Non-frail	17 (5.9)	0 (0.0)	0.092
Pre-frail	62 (21.6)	6 (13.6)	
Frail	208 (72.5)	38 (86.4)	
Group^*^			0.132
Non-HF & non-frail	79 (27.5)	6 (13.6)	
Non-HF & frail	144 (50.2)	25 (56.8)	
HF & frail	64 (22.3)	13 (29.5)	
**Laboratory data**			
NT-proBNP, pg/mL	1,897.5 (643.0–8,712.8)	2,735.0 (786.8–11,662.5)	0.405
LDL, mg/dL	97.0 (80.0–120.0)	85.0 (68.0–106.0)	0.017
Albumin, g/dL	4.0 (3.5–4.2)	3.5 (3.1–3.8)	<0.001
Hba1c, %	6.2 (5.7–7.0)	6.2 (5.5–7.0)	0.429
eGFR, ml/min per 1.73 m^2^	44.2 (32.5–52.4)	32.9 (17.7–45.4)	<0.001
Proteinuria, mg/g	0.17 (0.10–0.42)	0.15 (0.07–0.39)	0.271
**Medications**			
Diuretics	208 (72.5)	43 (97.7)	0.001
MRA	61 (21.3)	19 (43.2)	0.003
β-blocker	173 (60.3)	36 (81.8)	0.010
ACEI or ARB	213 (74.2)	36 (81.8)	0.368
Anti-platelet agents	196 (68.3)	30 (68.2)	1.000
Anti-coagulants	49 (17.1)	13 (29.5)	0.077
Digoxin	32 (11.1)	13 (29.5)	0.002

* *Frailty and non-frail older patients were classified according to the Rockwood frailty index. Continuous data were expressed as median (IQR) and analyzed by the Mann–Whitney U-test. Categorical data were expressed as number and percentage and analyzed by the Chi-Square test. CKD, chronic kidney disease; HF, heart failure; BMI, body mass index; CCI, Charlson Comorbidity Index; HFpEF, heart failure with preserved ejection fraction; HFrEF, heart failure with reduced ejection fraction; LVEF, left ventricular ejection fraction; MNA-SF, mini-nutritional assessment-short form; TUG, Timed Up-and-Go test; HGS, handgrip strength; NT-proBNP, N-terminal pro-B-type natriuretic peptide; LDL, low density lipoprotein; Hba1c, glycated hemoglobin; eGFR, estimated glomerular filtration rate; MRA, mineralocorticoid receptor antagonist; ACEI, angiotensin-converting enzyme inhibitor; ARB, angiotensin II receptor blockers; eGFR, calculated by using modified Modification diet of renal disease (MDRD) formula, was utilized to evaluate renal function*.

**Table 5:** Predictors of all-cause mortality in older CKD adults.

	**Univariate model**	**Model 1**	**Model 2**	**Model 3**
	**HR (95% CI)**	**HR (95% CI)**	**HR (95% CI)**	**HR (95% CI)**
Age	1.01 (0.96–1.06)	1.06 (0.96–1.17)	1.06 (0.96–1.17)	1.02 (0.97–1.08)
Male vs. Female	0.88 (0.46–1.69)	1.15 (0.28–4.77)	0.87 (0.21–3.50)	0.71 (0.35–1.42)
Heart failure	1.42 (0.74–2.72)			
Non-HF	Ref. –	Ref. –	Ref. –	Ref. –
HFpEF	1.28 (0.56–2.91)	1.48 (0.31–7.00)	1.27 (0.26–6.09)	0.54 (0.21–1.41)
HFrEF	1.63 (0.68–3.92)	4.78 (1.26–18.11)^*^	3.66 (0.92–14.51)	0.56 (0.19–1.67)
**CKD**				
Stage 3A	Ref. –	Ref. –	ref. –	Ref. –
Stage 3B	1.53 (0.68–3.40)	1.17 (0.39–3.47)	0.95 (0.32–2.85)	1.35 (0.60–3.03)
Stage 4	2.63 (1.16–5.96)^*^	1.46 (0.36–5.91)	1.06 (0.27–4.27)	2.03 (0.87–4.73)
Stage 5	4.22 (1.77–10.03)^**^	0.94 (0.11–8.20)	0.87 (0.10–7.69)	3.84 (1.51–9.75)^**^
CCI	1.17 (0.95–1.43)			
**Geriatric assessment**				
MNA-SF	0.92 (0.81–1.05)			
TUG, s	1.01 (0.99–1.04)			
TUG ≥24, s	1.60 (0.86–2.99)			
HGS, kg	0.92 (0.86–0.99)^*^	0.91 (0.84–0.99)^*^		
**HGS, kg**				
F >15.435/M >20.4	ref. –		ref. –	
F ≤ 15.435/M ≤ 20.4	4.01 (1.42–11.34)^**^		3.47 (1.15–10.42)^*^	
Rockwood frailty index	1.04 (1.02–1.06)^**^			1.04 (1.01–1.07)^**^

* *p < 0.05;*

** *p < 0.001; Model 1: The Cox proportional hazards model was used to evaluate the association of all-cause mortality with multivariate analysis among different stages of chronic kidney disease (CKD), the severity of heart failure (HF), and continuous levels of handgrip strength (HGS) in older adults. Model 2: The Cox proportional hazards model was used to evaluate the association of all-cause mortality with multivariate analysis among different stages of CKD, the severity of HF, and categorized HGS in older adults. Model 3: The Cox proportional hazards model was used to evaluate the association of all-cause mortality with multivariate analysis among different stages of CKD, the severity of HF, and Rockwood frailty index in older adults. All multivariate analysis was adjusted for age and gender. HFpEF, heart failure with preserved ejection fraction; HFrEF, heart failure with reduced ejection fraction; CCI, Charlson Comorbidity Index; MNA-SF, mini-nutritional assessment-short form; TUG, Timed Up-and-Go test; HGS, handgrip strength*.

### Survival Curves in the Subgroup Analyses Among HFpEF, HFrEF, and Different Levels of HGS, TUG, and 6MW in Patients With CKD

**Figure 2:**
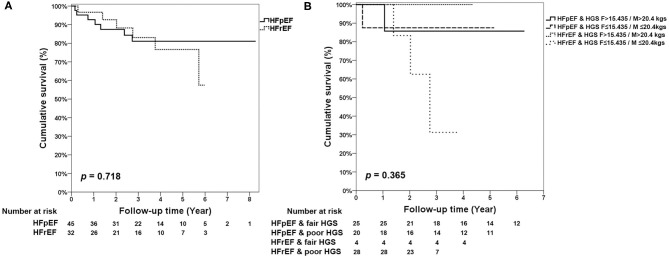
KM survival curves for **(A)** HFpEF and HFrEF. **(B)** Mortality stratified by the different levels of HGS, HFpEF, and HFrEF.

The KM survival curves showed no difference between HFpEF and HFrEF (*p* = 0.718) in the follow-up period (Figure 2A). In addition, there were also no composite effects of HFpEF and HFrEF with fair or poor HGS (*p* = 0.365; Figure 2B), short or long TUG (Supplementary Figure 2A), and 6MW (Supplementary Figure 2B) in old patients with CKD.

## Discussion

The principal findings on this retrospective cohort of old patients with CKD are their probably high prevalence of HF, impaired physical performance, and frailty, and mortality is significantly associated with low HGS, high Rockwood frailty score, and HF with reduced LVEF. The mortality is especially high among individuals with both HF and poor HGS.

In our study, we found that HGS was directly associated with survival in CKD patients, which was compatible with a previous study demonstrating that HGS is an independent predictor of composite renal outcomes in non-dialysis-dependent CKD (CKD-ND) patients (28). HGS, an indicator of frailty, has been used to approximate overall muscle function, particularly in patients with impaired tolerance of physical exertion and in hospitalized, deconditioned patients (29). Several potential mechanisms were speculated to explain the association between low HGS and poor outcomes in CKD patients. Lower HGS may represent underlying chronic systemic inflammation that may worsen patient outcomes by aggravating CVD and arterial stiffness and increasing susceptibility to infection (30). Besides this, the inflammation may be in association with acidosis, vitamin D deficiency, and uremic toxins, which decreased insulin sensitivity, leading to subsequent muscle wasting (30). Further studies are needed to elucidate the exact mechanisms linking HGS and outcome in CKD.

The findings of lower serum albumin and LDL values were both significantly associated with patient outcomes in patients with CKD_1−5_ or CKD_3b−5_ in univariate analyses. A previous study reports that a serum albumin level <3.7 g/dL was independently associated with poor composite renal outcomes (predialysis mortality and end-stage renal disease) in patients with CKD-ND (31). As serum albumin is highly influenced by inflammatory status, it is possible that the predictability of low serum albumin levels reflected more prevalent comorbidities or acute illnesses. Observational studies among apparently healthy individuals or patients with pre-existing CVD have repeatedly demonstrated a roughly linear relationship between serum total and LDL-cholesterol (LDL-C) and risk of death from CVD (32). Among patients with CKD, however, this relationship is much less obvious. Low total and LDL-C levels were also associated with higher mortality in patients with moderate to advanced stages of CKD, who were not yet on dialysis (33). The inverse association between low total and LDL-C levels and mortality may be explained in part by the presence of the so-called malnutrition–inflammation syndrome (32). It is suggested that decision to initiate statin treatment in patients with CKD should focus on the underlying cardiovascular risk and malnutrition-inflammation status, not just the lipid profile.

In our study, it was shown that there was no difference in HF and NT-proBNP between the group's death rates, but there was a significant difference concerning the use of diuretics, mineralocorticoid receptor antagonist (MRA), and digoxin. In addition, death risk was increased in patients with advanced CKD associated with HFrEF, which was in line with previous studies, indicating that concurrent renal disease and HF are directly related to a worse prognosis. Because of the finding that there were higher mortality risks among MRA, diuretics, and digoxin in our study, death was similar to some previous study reports (34–36) and probably attributable to several reasons. MRA (e.g., Spironolactone) in conjunction with ACEI and ARB may increase the risk of hyperkalemia in association with subsequent hospital readmission for hyperkalemia and in-hospital death (37). Furthermore, MRA may aggravate the extent of acidosis, which further depresses cardiac contractility and worsens HF (38). Although loop diuretics can alleviate body fluid overload, they also reduce GFR with neurohormonal activation and electrolyte disturbances. Besides this, it may increase myocardial fibrosis, which may be associated with disease progression and poor prognosis of HF (36, 39). Digoxin is predominantly excreted by the kidneys, and in impaired renal function, its pharmacokinetics can be influenced, resulting in toxicity of nausea and vomiting, and exacerbation of CKD (35).

Our results show that death risk was increased in CKD older patients associated with HF, which corresponds to previous studies indicating that concurrent renal disease and HF are directly related to a worse prognosis (7). Cardiac and renal diseases share common vascular risk factors, including the hemodynamic interactions of the heart and kidney in HF, the impact of atherosclerotic disease across both organ systems, neurohormonal activation, cytokines, the biochemical perturbations across the anemia–inflammation–bone mineral axis in CKD, and structural changes in the heart unique to kidney disease progression. The term cardiorenal syndrome (CRS) has been used to define different clinical conditions in which heart and kidney dysfunction overlap (40, 41), among which the classification of type 2 CRS is characterized by chronic abnormalities in cardiac function leading to kidney injury or dysfunction, and type 4 is characterized by cardiovascular involvement in patients affected by CKD at any stage. In CKD, it has been proposed that risks for HF include factors that affect preload and afterload, cardiomyopathic factors including left ventricular (LV) hypertrophy and fibrosis, and load-independent factors (neurohormonal activation, impaired iron utilization, anemia, demand ischemia, profibrotic factors, and inflammation) (42). However, our study was limited in the causes, and time sequences of CKD and CHF were not determined, and we were unable to examine which of the two disease processes was primary vs. secondary. Therefore, additional prospective studies to determine the temporal profile/change to both kidney and cardiac function over time with risk identification are necessary.

The diagnosis of HFrEF in the population with non-dialysis CKD parallels that of the population without CKD. The diagnosis of HFpEF in patients with non-dialysis CKD is difficult and should be supported by multiple objective measures, including HF symptoms and signs, typical clinical demographics, diagnostic laboratory tests, electrocardiogram, echocardiography, and function testing with exercise (16). The literature concerning mortality in HFpEF and CKD is inconsistent. Our study found that CKD was associated with a little higher mortality in HFrEF than in HFpEF, which was in line with previous studies, showing a lower mortality rate and a lower association between CKD and death in patients with HFpEF (43, 44). However, some studies report that, in CKD, HFpEF was a more powerful predictor of death than in HFrEF (45). As for reasons for the different prognosis of CKD with HFpEF and HFrEF, it was speculated that reduced EF may be associated with more advanced CKD with sympathetic and neurohormonal activation, which contributes to further renal deterioration (46), whereas in HFpEF, it may be due to endothelial dysfunction and inflammation leading to both cardiac and renal fibrosis (47, 48) and/or only reflections of the greater age and comorbidity burden.

In old CKD patients, the additional impacts of frailty and HF on prognosis were poorly known. Using the multivariate Cox proportional hazards analysis, we found that HFrEF combined with HGS resulting in severe or profound core activity limitation was associated with all-cause death in old CKD patients although TUG and 6MW insignificantly predicted mortality. As functional frailty was common with heterogeneity in old CKD populations, and it had prognostic implications, a joint evaluation on both physical function and HF should be necessary to predict patient outcomes more accurately.

The limitations of our study are as follows. First, this is a retrospective study. Therefore, longitudinal and prospective analyses are needed to further determine the effects of the physical decline associated with HF in different severity on mortality in these patients. Second, although the diagnosis of HFrEF was less debatable in this study, the reliability of the HFpEF diagnosis still needs further confirmation (15, 16). Third, the average cutoff values of TUG, HGS, and 6MW were set arbitrarily due to diverse physical function in different groups of patients with a major illness although reasonable for statistical analysis. Further studies on multimorbidity, drug history, lifestyle, or habits of a minimum volume of exercise can help to clarify these issues. Finally, the study was limited in that we did not have the direct causes of death, and knowing the causes of death may help better understand the reciprocal relationship between CKD, HF, and frailty in old patients.

In conclusion, frailty and CHF were two common conditions in old patients with CKD, and the two were associated with CKD stages. Physical limitation, frailty, and HF all predicted prognosis. Further, the combination of HF and poor HGS identified patients with high mortality risk. Efforts should be made to identify relevant factors of frailty and HF in these patients for better management strategies to improve morbidity, mortality, and patient-reported outcomes.

## Data Availability Statement

The raw data supporting the conclusions of this article will be made available by the authors, without undue reservation.

## Ethics Statement

The studies involving human participants were reviewed and approved by Institutional Review Board of Taichung Veterans General Hospital. Written informed consent for participation was not required for this study in accordance with the national legislation and the institutional requirements.

## Author Contributions

S-CW, Y-CC, C-SL, and S-YL conceived the idea and designed the study. S-CW, Y-CC, C-YH, C-SL, and S-YL carried out the analyses. S-CW and S-YL written and revised the manuscript. D-CT and S-YL supervised the implementation of the study. All authors reviewed and approved the manuscript prior to submission.

## Conflict of Interest

The authors declare that the research was conducted in the absence of any commercial or financial relationships that could be construed as a potential conflict of interest.
