# Supplementary material for: Impacts of Heart Failure and Physical Performance on Long-Term Mortality in Old Patients With Chronic Kidney Disease
Source: Front Cardiovasc Med. 2021 Jun 4;8:680098. doi: 10.3389/fcvm.2021.680098 (PMC8212996; doi:10.3389/fcvm.2021.680098)
Supplement: Supplementary file 1 [file Data_Sheet_1.pdf]

Supplementary Data

Impacts of Heart Failure and Physical Performance on Long-Term Mortality in Old Patients With Chronic Kidney Disease

Shuo-Chun Weng[1,2], Yu-Chi Chen[3], Chiann-Yi Hsu[4], Chu-Sheng Lin[5], Der-Cherng Tarng[1,6,7,8] and Shih-Yi Lin[1,9]*

[1]Institute of Clinical Medicine, School of Medicine, College of Medicine, National Yang Ming Chiao Tung University, Taipei, Taiwan, [2]Division of Nephrology, Department of Internal Medicine, Center for Geriatrics and Gerontology, Taichung Veterans General Hospital, Taichung, Taiwan, [3]Institute of Clinical Nursing, College of Nursing, National Yang Ming Chiao Tung University, Taipei, Taiwan, [4]Biostatistics Task Force of Taichung Veterans General Hospital, Taichung, Taiwan, [5]Department of Family Medicine, Center for Geriatrics and Gerontology, Taichung Veterans General Hospital, Taichung, Taiwan, [6]Department and Institute of Physiology, National Yang Ming Chiao Tung University, Taipei, Taiwan, [7]Division of Nephrology, Department of Medicine, Taipei Veterans General Hospital, Taipei, Taiwan, [8]Center for Intelligent Drug Systems and Smart Bio-devices (IDS2B); Department of Biological Science and Technology, College of Biological Science and Technology, National Yang Ming Chiao Tung, Hsinchu, Taiwan, [9]Division of Endocrinology and Metabolism, Department of Internal Medicine, Center for Geriatrics and Gerontology, Taichung Veterans General Hospital, Taichung, Taiwan

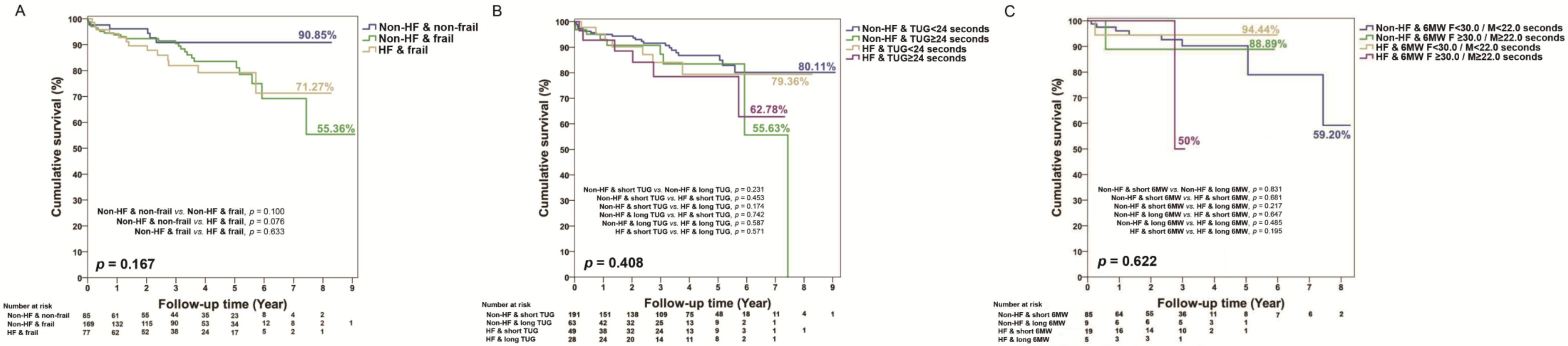

Supplementary Data 1. Kaplan-Meier survival curves for (A) non-heart failure (HF), HF, and frailty. (B) Mortality stratified by the different levels of timed up-and-go (TUG), HF, and non-HF. (C) Mortality stratified by the different levels of 6-meter walking test (6MW), HF, and non-HF.

A

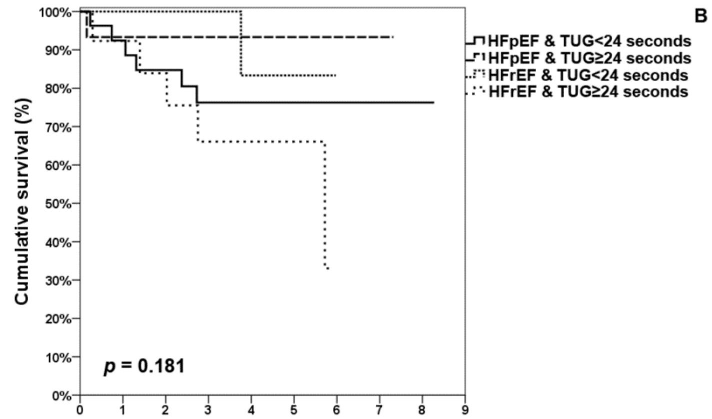

Number at risk

	0	1	2	3	4	5	6	7	8
HFpEF & short TUG	30	24	21	15	9	5	3	1	1
HFpEF & long TUG	15	12	10	7	5	5	2	1	
HFrEF & short TUG	19	14	11	9	4	4			
HFrEF & long TUG	13	12	10	7	6	3	1	0	

B

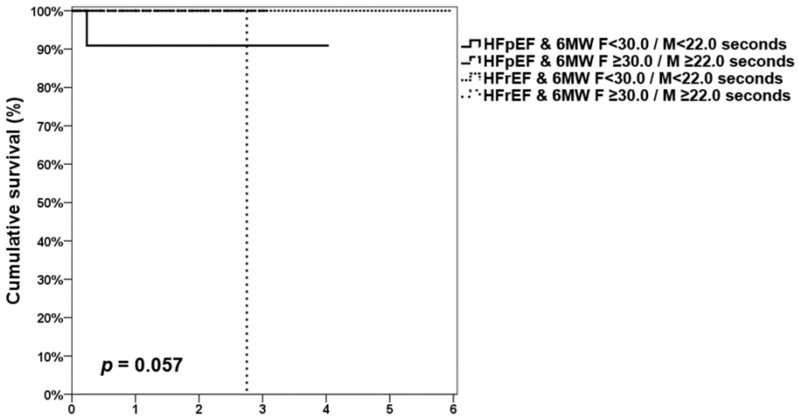

Number at risk

	0	1	2	3	4
HFpEF & short 6MW	34	31	28	25	23
HFpEF & long 6MW	11	11	11		
HFrEF & short 6MW	28	28	28	28	
HFrEF & long 6MW	4	4	4		

Supplementary Data 2. Kaplan-Meier survival curves for (A) different levels of timed up-and-go (TUG), HFpEF, and HFrEF. (B) Mortality stratified by the different levels of 6-meter walking test (6MW), HFpEF, and HFrEF.
